# Algorithm Analysis and Optimization of a Digital Image Correlation Method Using a Non-Probability Interval Multidimensional Parallelepiped Model

**DOI:** 10.3390/s24196460

**Published:** 2024-10-06

**Authors:** Xuedong Zhu, Jianhua Liu, Xiaohui Ao, Huanxiong Xia, Sihan Huang, Lijian Zhu, Xiaoqiang Li, Changlin Du

**Affiliations:** 1 School of Mechanical Engineering, Beijing Institute of Technology, Beijing 100081, China; 3120205243@bit.edu.cn (X.Z.); jeffliu@bit.edu.cn (J.L.); hxia@bit.edu.cn (H.X.); 2 Tangshan Research Institute, Beijing Institute of Technology, Tangshan 063015, China; hsh@bit.edu.cn; 3 Hebei Key Laboratory of Intelligent Assembly and Detection Technology, Tangshan 063000, China; 4 Shanghai Space Propulsion Technology Research Institute, Shanghai 201109, China; zljnpu@163.com (L.Z.); lxq826088571@163.com (X.L.); duchanglin0825@163.com (C.D.)

**Keywords:** uncertainty analysis, digital image correlation, optimization, parameter interval, reliability index

## Abstract

Digital image correlation (DIC), a widely used non-contact measurement technique, often requires empirical tuning of several algorithmic parameters to strike a balance between computational accuracy and efficiency. This paper introduces a novel uncertainty analysis approach aimed at optimizing the parameter intervals of a DIC algorithm. Specifically, the method leverages the inverse compositional Gauss–Newton algorithm combined with a prediction-correction scheme (IC-GN-PC), considering three critical parameters as interval variables. Uncertainty analysis is conducted using a non-probabilistic interval-based multidimensional parallelepiped model, where accuracy and efficiency serve as the reliability indexes. To achieve both high computational accuracy and efficiency, these two reliability indexes are simultaneously improved by optimizing the chosen parameter intervals. The optimized algorithm parameters are subsequently tested and validated through two case studies. The proposed method can be generalized to enhance multiple aspects of an algorithm’s performance by optimizing the relevant parameter intervals.

## 1. Introduction

Digital image correlation (DIC) is widely applied in various fields [1,2,3], including material mechanics, engineering inspection, biomedical sciences, and aerospace, to measure and study parameters such as deformation, displacement, and strain. DIC has achieved multiscale measurements from macroscopic to micro/nano scales [4,5,6,7], making it a versatile tool for researchers. Numerous endeavors have been dedicated to enhancing the accuracy of DIC algorithms; however, it is still a challenge to achieve both excellent calculation accuracy and efficiency. Moreover, there are usually a large number of input parameters that greatly impact the performances of a DIC algorithm and involve complex relationships, leading to difficulty in finding good algorithm parameters via engineering experience and trial and error. Therefore, an approach is needed to rapidly determine the input parameters for those algorithms to achieve both excellent calculation accuracy and efficiency.

Numerous studies have shown that many factors significantly influence the accuracy of DIC methods [8,9,10,11], such as computational parameters [12,13], subpixel registration algorithms and interpolation schemes [14,15,16,17,18], camera resolution [19], and testing environments, among others. However, there are no specific rules for setting the internal parameters of the algorithm, and the final parameter combination is typically determined based on engineering experience. Moreover, the impact of multi-source parameters on DIC calculation results is not yet clear, and it is crucial to understand the underlying patterns. This knowledge is of great significance for users to efficiently and rapidly determine and modify algorithm parameters, and it represents an important challenge in promoting the engineering application of DIC technology. Many domestic and foreign researchers have conducted studies on optimizing DIC methods. Goulmy et al. [20] presented a method to determine the image quality quickly and accurately according to the average intensity gradient of the speckle pattern standard and the standard deviation of the displacement on the image. Compared with the complete DIC calculation, this method can save a lot of time and optimize DIC parameters. Gao et al. [21] proposed a super-resolution (SR) reconstruction measurement method combined with three-dimensional digital image correlation of projection spots. At the same time, the DIC measurement system algorithm is optimized too, and the speckle-specific dual-mode prior adapted to the speckle image is proposed. The full-field displacement measurement experiment shows that the displacement error range can be reduced from 8 µm to 2 µm by using SR technology when the magnification and spot size are appropriate.

Recently, many applications of DIC have emerged, requiring methods and tools to evaluate and optimize those algorithms and techniques. The latest advances in experimental simulations have facilitated the estimation of measurement procedure accuracy [22,23]. These technologies also effectively optimize the DIC experimental device and improve the application rate of DIC. Typically, experimental optimization relies on approximate models of uncertainty [24]. It also leads to conjecture regarding whether the algorithm can be optimized by the uncertainty analysis model. Therefore, we focus on the non-probabilistic method based on interval variables [25,26] and believe that it has the potential to solve the above problems. As one of the most important non-probabilistic analysis methods, convex set theory [27,28,29,30] has been well developed. Among the available options, the multidimensional ellipsoid model [31,32] and the multidimensional parallelepiped model [33,34,35] are widely used in many fields [36,37,38,39]. Yang et al. [40] introduced an advanced optimal control strategy for spacecraft attitude management, incorporating interval uncertainty via a linear quadratic regulator (LQR) framework. Jiang et al. [41] introduced the bivariate subinterval method (BSM) to solve the multi-source uncertainty interval dynamic problem in mechanical systems. The findings indicate that, in comparison to the established bivariate Chebyshev method, the BSM achieves a superior balance between result accuracy and computational efficiency. Moreover, it proves to be more adept at analyzing interval dynamic problems within mechanical systems characterized by significant uncertainty dimensions and minor uncertainty levels.

This paper innovatively introduces a non-probabilistic uncertainty analysis model to optimize the parameters (including iteration steps, search range, and subset size) of a DIC algorithm, our previously proposed IC-GN-PC algorithm [42], to improve both computational efficiency and accuracy. Initially, the algorithm parameters’ uncertainty is characterized using a multidimensional parallelepiped model (MP-II). Subsequently, employing the IC-GN-PC algorithm, the strain in samples from the 2D-DIC Challenger dataset [43] is computed, facilitating the determination of both the peak signal-to-noise ratio (PSNR) and computational time. The surrogate model between the algorithm parameters and target indexes is established by using three-dimensional B-spline basis functions. Finally, the algorithm parameters are optimized by the reliability analysis method, and the optimized parameters are verified by the above data set. The presented method can be extended to enhance multiple performances of an algorithm by optimizing the interested parameter intervals.

## 2. Theory and Methodology

This section first introduces the IC-GN-PC algorithm structure and its evaluation criteria, then introduces the MP-II model and its reliability analysis method, and finally proposes a parameter optimization algorithm based on reliability.

### 2.1. IC-GN-PC Method

**Figure 1:**
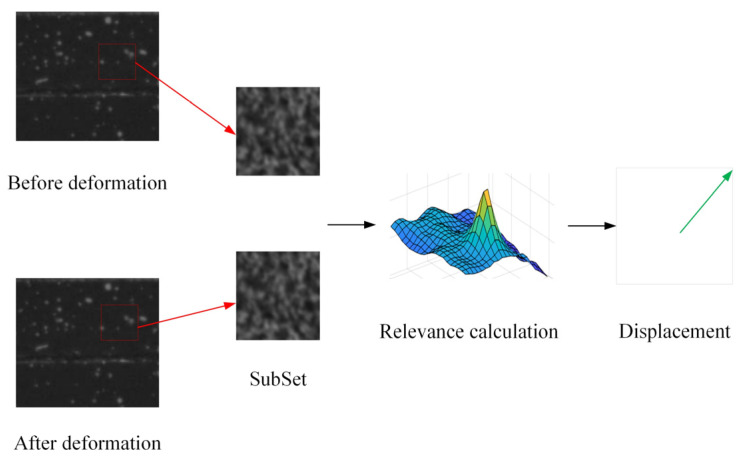
A schematic of DIC principle.

Digital image correlation (DIC) is a non-contact measurement technique employed for quantifying surface displacements pre- and post-deformation. The fundamental principle, illustrated in Figure 1, involves partitioning the image into subsets, identifying areas with the highest correlation before and after deformation, and subsequently calculating displacements. The key steps encompass shape function selection, cross-correlation function choice, integer pixel estimation, subpixel iteration, and related procedures.

A first-order shape function characterizes a range of deformations, including translation, expansion, rotation, shear, and their combinations within the deformation subset. The expression is as follows:

(1) $$\{\begin{array}{c} x^{\prime }=x+u+\frac{\partial u}{\partial x}\Delta x+\frac{\partial u}{\partial y}\Delta y\\ y^{\prime }=y+v+\frac{\partial v}{\partial x}\Delta x+\frac{\partial v}{\partial y}\Delta y \end{array}$$

Here, (*x*, *y*), (*x′*, *y′*) denote the coordinates of any point within the reference subset prior to deformation and its respective post-deformation counterpart. $u,\;v,\;\frac{\partial u}{\partial x},\;\frac{\partial u}{\partial y},\;\frac{\partial v}{\partial x},\;\frac{\partial v}{\partial y}$ signifies both the displacement of the subset’s central point and the first-order derivative of this displacement.

During the integer pixel stage, an efficient prediction-correction integer pixel positioning method is devised to enhance accuracy and prevent convergence to local optima. Initially, the normalized least square distance with zero mean is chosen as the search correlation function:

(2) $$C={\sum_{i=1}^{MX}\sum_{j=1}^{MY}\left[\frac{f(x,y)-\bar{f}}{\sqrt{\sum_{i=1}^{MX}\sum_{j=1}^{MY}{\left(f(x,y)-\bar{f}\right)}^{2}}}-\frac{g(x^{\prime },y^{\prime })-\bar{g}}{\sqrt{\sum_{i=1}^{MX}\sum_{j=1}^{MY}{\left(g(x^{\prime },y^{\prime })-\bar{g}\right)}^{2}}}\right]}^{2}$$

Here, *C* denotes the cross-correlation coefficient, while *MX* and *MY* refer to the dimensions of the detection window. The variables *f* and *g* represent the image grayscale values within the detection window before deformation (reference subset) and after deformation (target subset), respectively. The upper horizontal line signifies the average gray value within the detection window. Optimal subset similarity is achieved when the function value is 0, while maximum dissimilarity occurs when the function value is 1.

**Figure 2:**
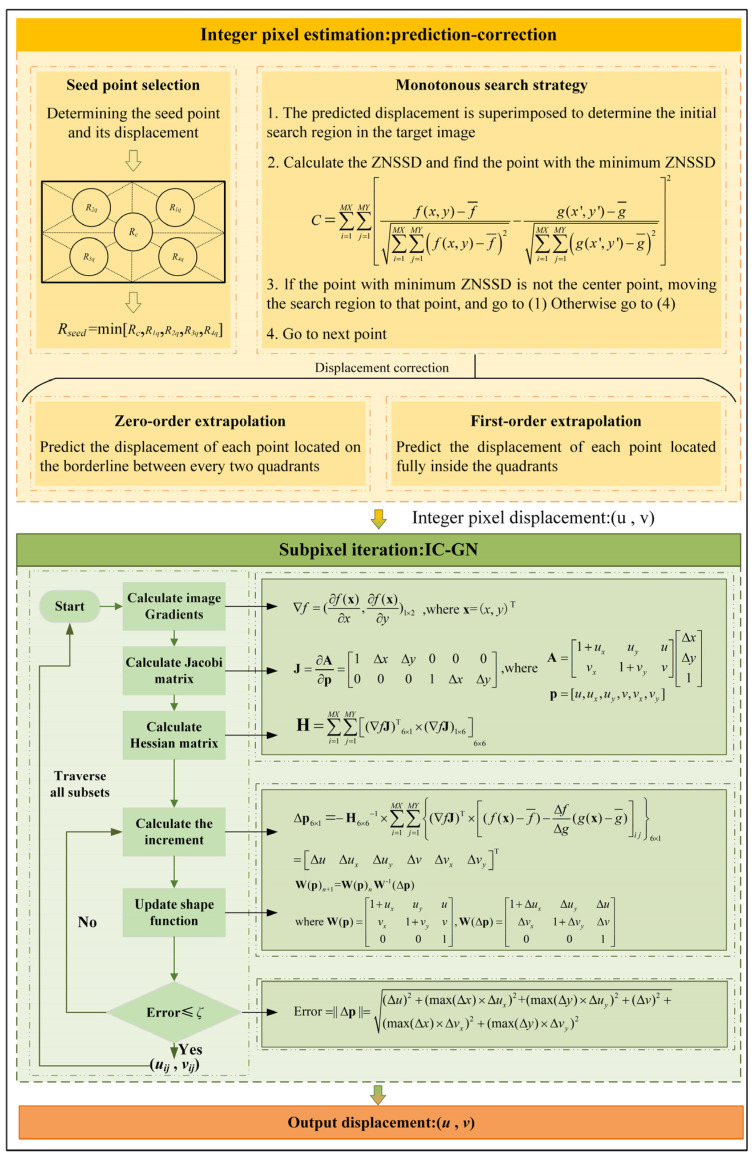
A flowchart of the IC-GN-PC algorithm.

Figure 2 shows the flowchart of the IC-GN-PC method, including two procedures: integer pixel estimation, followed by subpixel iteration. The integer pixel estimation applies the prediction-correction algorithm, which mainly includes the selection of seed points and the prediction and correction of displacement (please see reference [42] for the detailed prediction-correction algorithm). The subpixel iteration applies the IC-GN algorithm, which mainly includes constant pre-calculation, iterative computation, and convergence check in every subset, then traverses all subsets (please see reference [44] for the detailed IC-GN algorithm).

### 2.2. Non-Probabilistic Reliability Analysis

The reliability analysis of the IC-GN-PC method’s algorithm parameters encompasses three key phases: parameter uncertainty characterization, uncertainty transfer, and conducting the reliability analysis itself. Initially, the MP-II model is applied to articulate the algorithm parameters’ uncertainty. Following this, uncertainties are conveyed through a surrogate model constructed with B-spline basis functions. The process culminates in a non-probabilistic reliability analysis, which calculates the reliability index.

The uncertainty domain expression of MP-II model is:

(3) $$\Omega_{X}^{P.n}=\left\{X|\left|G_{P}(X-X^{m})\right|\leq e\right\}$$

**Figure 3:**
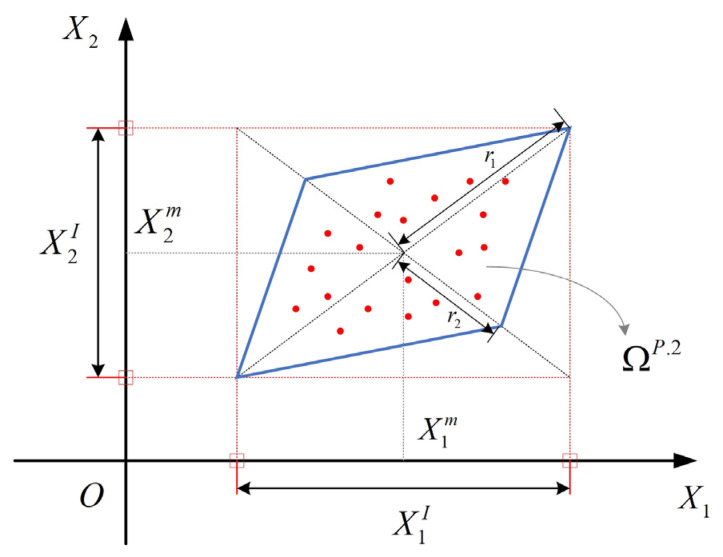
The correlation measure of interval variables in MP-II model.

*X_i_*∈*X_i_^I^* = [*X_i_^L^*, *X_i_^U^*], *I* = 1, 2,… *n*, is the algorithm parameter intervals, where *X_i_^U^* and *X_i_^L^* denote their upper and lower bounds. ***G_P_*** determines the configuration and dimensions of the uncertainty domain, which is called the characteristic matrix. ***X^m^*** is a vector of the corresponding interval midpoints. ***e*** = [1 1 1 … 1] is a n-dimensional vector. For any two interval variables *X*_1_ and *X*_2_, their correlation is quantitatively described using a parallelogram, Ω*^P^*^.2^, as shown in Figure 3.

The correlation coefficient of *X*_1_ and *X*_2_ is defined as:

(4) $$\rho_{X_{1}^{I}X_{2}^{I}}=\frac{Cov(X_{1},X_{2})}{X_{1}^{r}X_{2}^{r}}=\frac{r_{1}^{2}-r_{2}^{2}}{r_{1}^{2}+r_{2}^{2}}$$

Here, *r*_1_ and *r*_2_ are the semi-axis length of *X*_1_ in direct and inverse proportions to *X*_2_, respectively, and $X_{1}^{r}$ and $X_{2}^{r}$ denote the interval radii of *X*_1_ and *X*_2_, respectively. For a multidimensional problem, the uncertainty characterization is defined as:

(5) $$\begin{array}{l} G_{P}={\left(diag\left[\begin{array}{cccc} X_{1}^{r} & X_{2}^{r} & \ldots & X_{n}^{r} \end{array}\right]S\right)}^{-1}\\ S=TH\\ T=diag\left[\begin{array}{cccc} w_{1} & w_{2} & \ldots & w_{n} \end{array}\right]\\ w_{i}=\frac{1}{\sum_{j=1}^{n}\left|H\left(i,j\right)\right|},i=1,2,\ldots ,n \end{array}$$

Here, ***S*** is the shape matrix and ***H*** is the square root matrix of ***ρ***.

(6) $$H=\rho^{1/2}={\left[\begin{array}{cccc} \rho_{X_{1}^{I}X_{1}^{I}} & \rho_{X_{1}^{I}X_{2}^{I}} & \cdots & \rho_{X_{1}^{I}X_{n}^{I}}\\ \rho_{X_{2}^{I}X_{1}^{I}} & \rho_{X_{2}^{I}X_{2}^{I}} & \cdots & \rho_{X_{2}^{I}X_{n}^{I}}\\ \vdots & \vdots & \ddots & \vdots \\ \rho_{X_{n}^{I}X_{1}^{I}} & \rho_{X_{n}^{I}X_{2}^{I}} & \cdots & \rho_{X_{n}^{I}X_{n}^{I}} \end{array}\right]}^{1/2}$$

Through the implementation of the IC-GN-PC method outlined in Section 2.1, we can extract PSNR and computational time data corresponding to various algorithm parameters. The uncertainty transfer from the selected algorithm parameter intervals to PSNR and computational time is elucidated through a surrogate model. The B-spline basis-function fitting is represented as:

(7) $$\phi \left(x\right)\approx \sum_{j=1}^{m}a_{j}N_{j}^{k}\left(x\right)=N_{j}^{k}\left(x\right)a$$

where **N***_j_^k^*(*x*) is the B-spline basis functions and **a** is a vector of the corresponding weight. A least square method is applied to minimize the fitting error as:

(8) $$\begin{array}{l} E=\frac{1}{2}{\left[\phi \left(x_{i}\right)-N_{j}^{k}\left(x_{i}\right)a\right]}^{T}\left[\phi \left(x_{i}\right)-N_{j}^{k}\left(x_{i}\right)a\right]\;\;i=1,2,\ldots ,n\\ \frac{\partial E}{\partial a}=-{\left[N_{j}^{k}\left(x_{i}\right)\right]}^{T}\left[\phi \left(x_{i}\right)-N_{j}^{k}\left(x_{i}\right)a\right]=0 \end{array}$$

The purpose of reliability analysis is to quantitatively describe the reliability of existing algorithm parameters under the critical state requirements of a given PSNR and computation time. These two correspond to precision reliability and efficiency reliability, respectively, thus the two critical-state functions can be expressed as:

(9) $$\left\{ \begin{array}{l} c_{P}\left(X\right)=PSNR^{*}-D_{P}\\ PSNR^{*}=N_{slj}^{T}\left(X\right)a_{P},X\in \Omega_{X}^{P.n} \end{array}\right.$$

(10) $$\left\{ \begin{array}{l} c_{T}\left(X\right)=D_{T}-T^{*}\\ T^{*}=N_{slj}^{T}\left(X\right)a_{T},X\in \Omega_{X}^{P.n} \end{array}\right.$$

Here, *c_P_*(***X***) and *c_T_*(***X***) represent the critical-state function of PSNR and computational time respectively. PSNR* and T* are given by the corresponding B-spline surrogate models, and *D_P_* and *D_T_* represent the requirements of PSNR and computational time, respectively, which can be set according to different requirements for accuracy and efficiency. Generally speaking, *c*(***X***) ≥ 0 means that the current algorithm parameters satisfy the criteria for both accuracy and efficiency, otherwise not.

**Figure 4:**
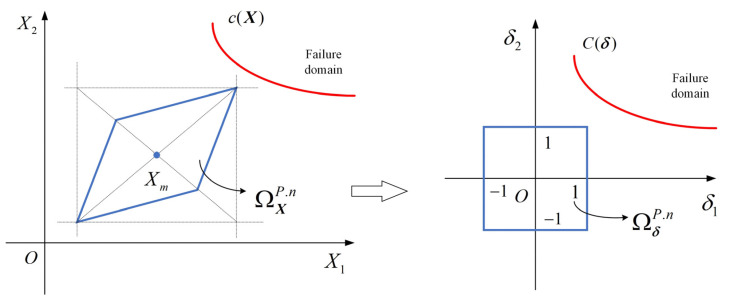
Standardization from ***X*** space to ***δ*** space.

The reliability is evaluated in a so-called standardized ***δ*** space, where the uncertainty domain is a multidimensional unit cube, as illustrated in Figure 4. The transformation process is defined as:

(11) $$X=G_{P}^{-1}\delta +X^{m}$$

The reliability index is computed within the ***δ*** space, characterized as the shortest infinite-norm distance from the ***δ*** space ‘s origin O, to *C*(***δ***). To find the shortest infinite-norm distance, we apply an optimization method:

(12) $$\left\{ \begin{array}{l} OBJ:\;\eta^{P}=\mathrm{sign}(c(X^{m}))\underset{\delta }{\min}{\left\|\delta \right\|}_{\infty }\\ \begin{array}{cc} & \;\;\;= \end{array}\mathrm{sign}(c(X^{m}))\min\left\{\max\left(\left|\delta_{1}\right|,\left|\delta_{2}\right|,\cdots ,\left|\delta_{n}\right|\right)\right\}\\ \begin{array}{cc} s.t. & \end{array}\;C(\delta )=0 \end{array}\right.$$

**Figure 5:**
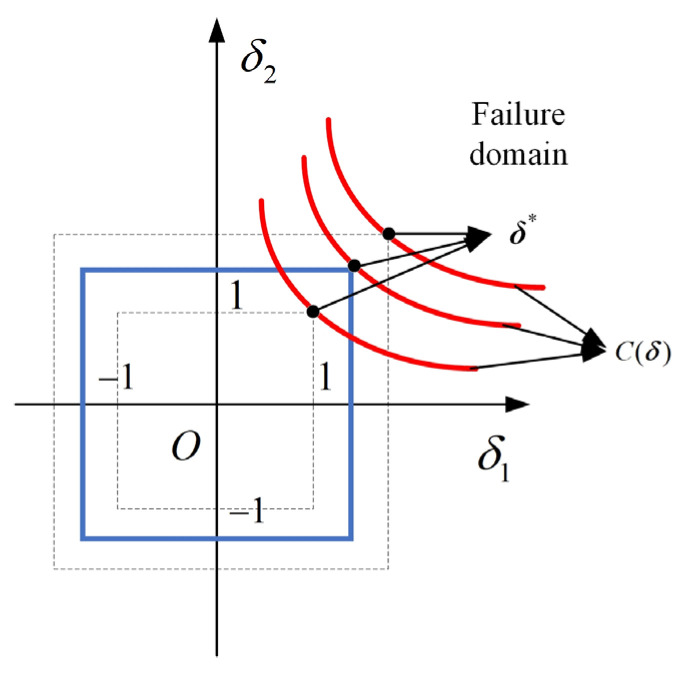
A schematic of three typical situations of the non-probabilistic reliability index.

There are three typical situations for the reliability index as shown in Figure 5: (1) *η^P^* > 1, the critical-state surface is away from the uncertainty domain, indicating that the requirements are perfectly satisfied; (2) *η^P^* = 1, indicating a just satisfaction; (3) *η^P^* < 1, implying a possible failure.

### 2.3. Parameter Interval Optimization

Supposing a case with *m* reliability indexes and *n* parameters, the parameter interval optimization problem is defined as:

(13) $$\begin{array}{l} \left\{ \begin{array}{l} OBJ:\;\min\left(\eta_{i}^{P}\right)|\eta_{i}^{P}\geq 1\\ s.t.\;\;X_{ki}^{U}-X_{ki}^{L}=2X_{k}^{r0},\;X_{ki}^{L}>0 \end{array}\right.\\ X_{k*}^{I}=\left[X_{k*}^{L},X_{k*}^{U}\right]=\Pi \left[X_{ki}^{L},X_{ki}^{U}\right],\;k=1,2,\ldots ,n;\;i=1,2,\ldots ,m \end{array}$$

where $\left[X_{k*}^{L},X_{k*}^{U}\right]$ is the optimized interval, represented by $X_{k*}^{I}$, and Π is the intersection operator. The core concept of the suggested optimization approach is to find the widest interval for each parameter so that all the reliability indexes reach minimum values but not less than 1. Notably, the constraint conditions presented in Equation (12) should be changed for different applications. For the instanced DIC algorithm, the constraint conditions are given as presented, where the radius of the parameter intervals remains at its initially given value $X_{k}^{r0}$ and all the intervals must be positive. The optimization process of parameter intervals can be summarized as follows:

1. Computing the *n* reliability indexes *η*_1_*^P^*, *η*_2_*^P^*, *… η_m_^P^* under corresponding reliability requirements according to Section 2.2;
2. Adjusting the interval median of a parameter so that the reliability index *η_i_^P^* reaches a minimum value but not less than 1. Repeating this operation for each reliability index, and obtaining the optimal interval of the parameter by taking the intersections of the aforementioned intervals. If all the reliability requirements cannot be satisfied for a parameter, then its optimal interval is empty;
3. Doing step (2) for all the parameters to obtain the optimized parameter intervals for the instanced algorithm.

## 3. Analysis and Discussion

To verify the proposed method, we test the IC-GN-PC algorithm using the speckle images from the 2D-DIC Challenger dataset [43]. The PSNR and computational time of the DIC algorithm are taken as the target performances, and three intervals of the parameters of subset size, search range, and iteration steps in the algorithm are optimized to improve the two target performances.

### 3.1. Presentation of the IC-GN-PC Method

**Figure 6:**
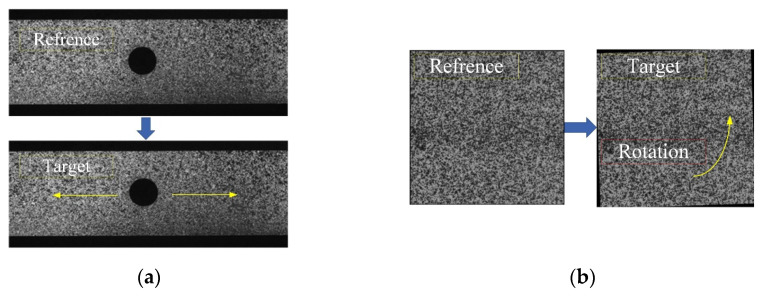
Reference and target images of two cases from the 2D-DIC Challenger dataset [43]. (**a**) Case 1 was obtained through the plate hole tensile test; (**b**) case 2 was generated using the rotating FFT method.

Here, we selected two cases of typical sample data. Figure 6a shows case 1 obtained through the plate hole tensile test, and Figure 6b shows case 2 generated using the rotating FFT method.

**Figure 7:**
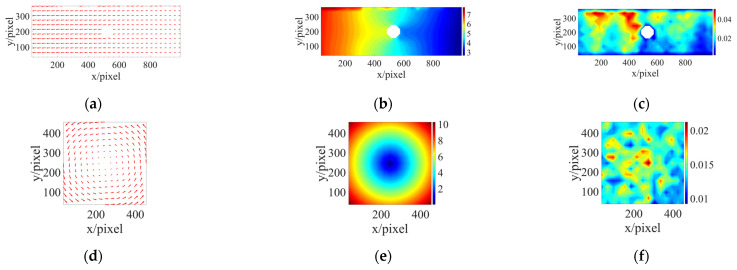
(**a**,**d**) Displacement vector fields; (**b**,**e**) pixel motions; (**c**,**f**) ZNSSD distributions of the two cases.

As shown in Figure 7, the results of the displacement vector field, the pixel motion detected by the DIC, and the correlation coefficient distribution of two samples were obtained using the IC-GN-PC method. These results are based on the parameter selection of the subset size, the search range, and iteration numbers, which are 30 × 30 pixels, ±20 pixels, and 20 steps, respectively. Figure 7c,f depict the distribution of ZNSSD correlation coefficients. In case 1 and case 2, the displacement vector field conforms to the corresponding actual situation, and the correlation coefficient of each point is less than 0.045. Among them, the maximum correlation coefficient of case 2 is less than 0.025. Under the above parameters, we determine that the PSNR of case 1 is 26.3792 with a computational time of 7.8866 s and the PSNR of case 2 is 27.7902 with a computational time of 4.4188 s. The aforementioned results demonstrate that the IC-GN-PC method can yield high-quality analysis results for the selected dataset, but under the action of multi-source parameters, and by fine-tuning the parameters, the algorithm’s calculation accuracy and computational efficiency can be further enhanced.

### 3.2. Reliability Analysis and Optimization of Parameter Intervals

The three selected algorithm parameters should be quantified as interval variables. The initially given intervals for the three parameters are shown in Table 1, and the data samples generated in those intervals using a uniform random algorithm are listed in Table 2.

According to Table 2 and Equation (5), the ***G****_P_* can be computed as follows:

(14) $$G_{P}=\left[\begin{array}{ccc} 0.173 & -0.031 & 0.046\\ -0.048 & 0.108 & -0.040\\ 0.056 & -0.031 & 0.144 \end{array}\right]$$

Thus, the MP-II uncertainty domain of the selected algorithm parameters is constructed and expressed as follows:

(15) $$\Omega^{P.3}=\left\{\left[\begin{array}{c} X_{1}\\ X_{2}\\ X_{3} \end{array}\right]|\left|{\left[\begin{array}{ccc} 0.173 & -0.031 & 0.046\\ -0.048 & 0.108 & -0.040\\ 0.056 & -0.031 & 0.144 \end{array}\right]}^{-1}\left[\begin{array}{c} X_{1}-30\\ X_{2}-25\\ X_{3}-18 \end{array}\right]\right|\leq \left[\begin{array}{c} 1\\ 1\\ 1 \end{array}\right]\right\}$$

According to the theory in Section 2.2, we sampled in the three algorithm parameter intervals, then implemented the IC-GN-PC algorithm to yield the corresponding data of PSNR and computational time. The surrogate models for case 1 and case 2 were respectively established by fitting those data using a B-spline basis function set with the order and number of the basis functions of 2 and 3. Finally, the B-spline surrogate models of PSNR and computational time were obtained, and their weight vectors for the two cases are listed in Table 3, Table 4, Table 5 and Table 6.

To verify the accuracy of the B-spline surrogate models, we carried out numerical tests on the aforementioned two cases with the data samples listed in Table 2, where the PSNR and computational time predicted by the surrogate models are compared with those by the IC-GN-PC algorithm. The computations are presented in Table 7 and Table 8. The maximum relative errors of PSNR are 1.65% and 1.90%, respectively, and those of computational time are 1.53% and 1.83% respectively. These indicate that the prediction accuracies of the surrogate models are very good, which is sufficient to meet the requirements of subsequent reliability analysis.

**Figure 8:**
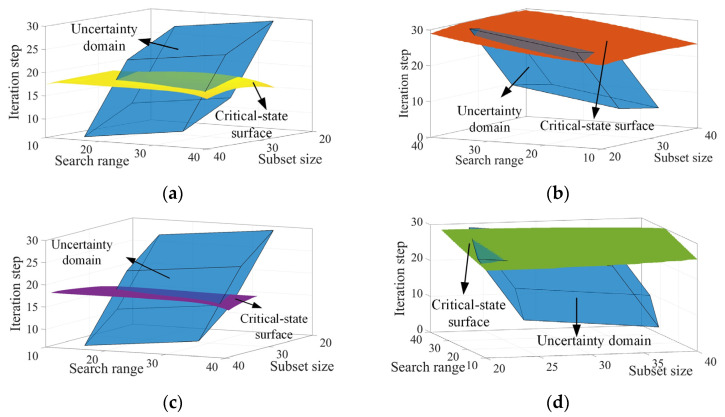
The uncertainty domain and four critical-state surfaces under the initially given parameter intervals. The hexahedron colored in blue in all the subfigures represents the uncertainty domain, and the surfaces colored in yellow (**a**) and red (**b**) represent the critical-state surfaces of PSNR and computational time of case 1, respectively, while the surfaces in purple (**c**) and green (**d**) represent those of case 2.

Before optimizing the parameters of the IC-GN-PC algorithm, the reliability indexes of accuracy and efficiency were calculated using the critical-state surfaces generated by the B-spline surrogate models. Firstly, we set the reliability requirements of accuracy and efficiency *D_P_* = 26.4, *D_T_* = 9 s, and *D_P_* = 26.4, *D_T_* = 6 s for cases 1 and 2, respectively. According to the reliability analysis of Section 2.2, the spatial relationships between the four critical-state surfaces and the uncertainty domain of the selected algorithm parameters for the two cases under the initially given parameter intervals were computed, and are presented in Figure 8. All the critical-state surfaces intersect with the uncertainty domain, and the corresponding non-probabilistic reliability indexes *η^P^*_P1_ = 0.78, *η^P^*_T1_ = 0.75, *η^P^*_P2_ = 0.52, *η^P^*_T2_ = 0.89. The indexes are less than 1, indicating that the initially given parameter intervals cannot completely meet the expected reliability requirements of PSNR and computational time.

**Figure 9:**
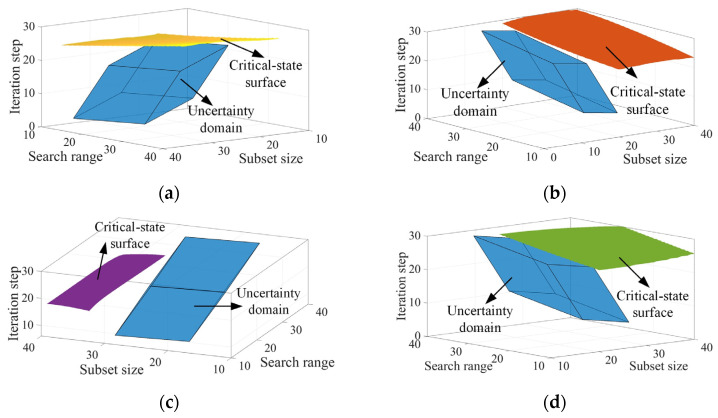
Four spatial states of uncertainty domain and critical-state surfaces under the optimized interval of the subset size. The hexahedron colored in blue in all the subfigures represents the uncertainty domain, and the surfaces colored in yellow (**a**) and red (**b**) represent the critical-state surfaces of PSNR and computational time of case 1, respectively, while the surfaces in purple (**c**) and green (**d**) represent those of case 2.

**Figure 10:**
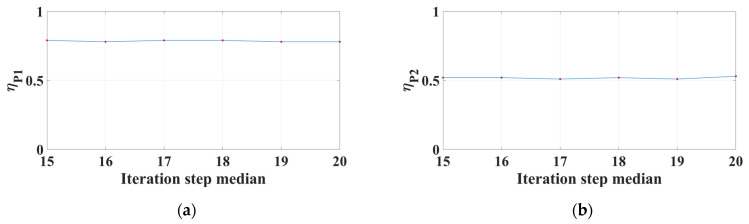
Reliability indexes of PSNR versus the interval step median for (**a**) case 1 and (**b**) case 2.

Next, we improve the reliability indexes of accuracy and efficiency to greater than 1 by optimizing the intervals of the three selected algorithm parameters. For the subset size, the non-probabilistic reliability indexes of PSNR and computational time are respectively improved to *η^P^*_P1_ = 1.06 and *η^P^*_T1_ = 1.02 for case 1 and to *η^P^*_P2_ = 1.04 and *η^P^*_T2_ = 1.01 for case 2 when the subset size interval is adjusted to [16, 36], [12, 30] for case 1 and [13, 33], [13, 33] for case 2. The results indicate that all the reliability requirements are satisfied. Taking the intersections of the adjusted intervals for each case, we have the optimized interval for the subset size of [16, 30] for case 1 and [13, 33] for case 2. The new geometric relationships under the optimized interval of the subset size are shown in Figure 9. For the search range, the reliability indexes *η^P^*_P1_ and *η^P^*_T1_ are respectively improved to 1.05 and 1.02 as the search range interval is adjusted to [2, 32] and [26, 56] for case 1, thus its optimal interval is [26, 32]. For case 2, the reliability index *η^P^*_T2_ is improved to 1.02 as the search range interval is adjusted to [16, 46]; however, the reliability index *η^P^*_P2_ only reaches a maximum value of 0.73, less than 1. As a consequence, the optimal interval is [16, 46] because the interval that cannot satisfy the reliability requirement is ignored. For the iteration step, it is found that the reliability indexes *η^P^*_P1_ and *η^P^*_P2_ in cases 1 and 2 are slightly impacted by the median value of the iteration step interval, as shown in Figure 10, thus the intervals in the two cases are both ignored. The optimal intervals of the iteration step are [3, 27] and [4, 28], yielding *η^P^*_T1_ = 1.03 and *η^P^*_T2_ = 1.08 for cases 1 and 2. The optimized results are summarized in Table 9.

To validate the optimization results, we tested the optimized intervals of the three selected parameters in the two cases. Here, we randomly sampled the optimized intervals to generate three sets of the selected parameters for each case, then ran the IC-GN-PC algorithm to output the PSNR and computational time. The data and results are summarized in Table 10. The values of PSNR yielded by the IC-GN-PC algorithm for the three sets in cases 1 and 2 are all greater than 26.4, and those of the computational time are all less than 9 s in case 1 and 6 s in case 2. These indicate that the specified accuracy and efficiency requirements for the IC-GN-PC algorithm are achievable within the optimized intervals.

## 4. Conclusions

This study introduces a non-probabilistic interval uncertainty method to analyze and optimize the performance of a DIC algorithm, focusing on the impact of three selected parameters on the algorithm’s computational accuracy and efficiency through reliability analysis. By optimizing the intervals of these parameters, the reliability indexes were significantly improved.

This paper innovatively believes that the fluctuation instability of the computational efficiency and accuracy of the DIC algorithm stems from the uncertainty of the internal parameters, and the method proposed in this paper is an important step in this kind of problem. The tested DIC algorithm in this paper is only an instance, of course, We posit that the presented method can be extended to analyze and enhance the multiple performances of other algorithms, thereby broadening its application scope. The presented method also has strong generalization ability, such as more complex input parameters and target indexes, and even such time-varying parameters. We also know that if this method can be applied to more practical engineering problems, it will be more mature and have engineering significance, which is also the direction of our follow-up efforts.

## Figures and Tables

**Table 1:** The uncertainty parameters of the selected algorithm parameters.

Selected Algorithm Parameters	Marginal Interval	Interval Median	Interval Radius
Subset size	[20, 40]	30	10
Search range	[10, 40]	25	15
Iteration steps	[6, 30]	18	12

**Table 2:** Data samples of the selected algorithm parameters.

Serial	Subset Size	Search Range	Iteration Steps
1	22	25	24
2	28	23	12
3	39	30	18
4	36	31	23
5	40	39	29
6	33	18	19
7	20	31	18
8	37	30	9
9	39	15	9
10	34	13	12
11	35	25	27
12	35	33	12
13	28	20	26
14	33	28	12
15	23	16	29

**Table 3:** Case 1: Weight vector ***p*_1_** of the surrogate model of PSNR.

***p***_1_(1:9)	29.79	24.81	27.69	26.04	27.10	25.86	27.61	26.00	25.93
***p***_1_(10:18)	25.83	23.72	21.27	26.82	29.36	26.85	26.57	26.87	24.38
***p***_1_(19:27)	24.65	22.54	26.82	23.45	25.41	24.89	25.72	27.95	26.23

**Table 4:** Case 2: Weight vector ***p*_2_** of the surrogate model of PSNR.

***p***_2_(1:9)	31.06	29.42	23.91	23.70	27.92	23.68	45.55	23.64	28.03
***p***_2_(10:18)	29.65	27.82	21.26	21.42	25.83	21.96	43.24	21.36	26.57
***p***_2_(19:27)	28.45	31.42	22.64	26.56	26.54	20.34	46.94	24.54	26.81

**Table 5:** Case 1: Weight vector ***t*_1_** of the surrogate model of computational time.

***t***_1_(1:9)	−0.41	−1.90	−1.52	−1.62	−1.41	−1.11	−0.79	−0.95	−1.62
***t***_1_(10:18)	7.93	7.31	9.28	6.64	6.76	5.14	6.07	4.28	7.35
***t***_1_(19:27)	17.64	14.92	20.87	14.55	14.35	11.31	12.22	9.36	14.25

**Table 6:** Case 2: Weight vector ***t*_2_** of the surrogate model of computational time.

***t***_2_(1:9)	−0.77	−0.73	−0.57	−0.70	−0.87	−0.78	−0.99	−0.47	−0.72
***t***_2_(10:18)	4.97	4.96	4.36	4.02	4.01	3.76	4.10	2.55	3.61
***t***_2_(19:27)	10.15	12.36	10.16	9.44	8.16	7.90	7.40	6.00	8.16

**Table 7:** Case 1: Prediction error.

Serial	PSNR of DIC	Predicted PSNR	PSNR Relative Error (%)	Computational Time of DIC	Predicted Time	Computational Time Relative Error (%)
1	26.9392	26.6837	0.95	8.2749	8.3711	1.16
2	26.8975	26.6194	1.03	4.5103	4.5795	1.53
3	26.1781	26.3895	0.80	5.8767	5.9538	1.31
4	26.8704	26.4790	1.45	7.8004	7.6921	1.38
5	26.3277	26.2404	0.33	9.1381	9.0499	1.01
6	26.5841	26.4656	0.44	7.3573	7.3795	0.30
7	26.4474	26.6595	0.80	6.1410	6.2133	1.17
8	26.5605	26.4556	0.39	3.4255	3.4233	0.06
9	26.2544	26.3843	0.49	3.6621	3.6962	0.93
10	26.2734	26.3359	0.23	4.9886	4.9128	1.51
11	26.0813	26.5140	1.65	9.6197	9.6565	0.38
12	26.7937	26.4904	1.13	4.0818	4.0763	0.13
13	26.8975	26.5856	1.15	8.8906	8.9476	0.64
14	26.5841	26.5646	0.07	4.9111	4.8519	1.05
15	26.7866	26.6796	0.39	10.5578	10.5490	0.08

**Table 8:** Case 2: Prediction error.

Serial	PSNR of DIC	Predicted PSNR	PSNR Relative error (%)	Computational Time of DIC	Predicted Time	Computational Time Relative Error (%)
1	26.6656	27.0654	1.49	5.5700	5.5069	1.13
2	25.2075	25.3182	0.43	2.7996	2.8250	0.90
3	27.0882	26.5753	1.90	3.7305	3.7123	0.48
4	24.9728	25.2890	1.26	4.6776	4.6315	0.98
5	24.2411	24.5181	1.14	4.9305	4.9775	0.85
6	28.4261	28.1620	0.93	4.4975	4.4672	0.67
7	31.0711	30.9611	0.35	3.9062	3.8675	0.98
8	24.5835	25.0092	1.73	2.2204	2.1918	1.28
9	26.5893	26.3208	1.01	2.4378	2.4121	1.05
10	27.4022	27.2739	0.46	3.2079	3.1819	0.81
11	27.6861	27.6310	0.19	5.9777	5.8887	1.48
12	25.9449	26.3268	1.47	2.4908	2.5233	1.30
13	25.2075	25.6269	1.66	5.6671	5.7626	1.68
14	25.6900	25.7379	0.18	2.6208	2.6688	1.83
15	25.5279	25.8053	1.08	6.4818	6.5849	1.59

**Table 9:** Optimization results of the selected algorithm parameter intervals.

Selected Algorithm Parameters	Case 1				Case 2			
	Optimized Interval	*η^P^*_P1_	Optimized Interval	*η^P^*_T1_	Optimized Interval	*η^P^*_P2_	Optimized Interval	*η^P^*_T2_
**Subset size**	[16, 36]	1.06	[12, 30]	1.02	[13, 33]	1.04	[13, 33]	1.01
**Search range**	[2, 32]	1.05	[26, 56]	1.02	[0, 30] *	0.73	[16, 46]	1.02
**Iteration step**	**	0.79	[3, 27]	1.03	**	0.53	[4, 28]	1.08

* The optimized interval of the search range can yield a maximum *η^P^*_P2_ but less than 1, thus this optimized interval should be ignored in the intersection operations. ** The interval of the iteration step slightly impacts the reliability indexes *η^P^*_P1_ and *η^P^*_P2_ and their values are always less than 1, thus this interval should be ignored in the intersection operations.

**Table 10:** Tested samples and results of the method validation.

Case	Sample	Subset Size	Search Range	Iteration Steps	PSNR	Computational Time
Case 1	1	22	27	15	26.80	5.54 s
	2	18	26	21	26.63	7.85 s
	3	27	30	18	26.73	6.32 s
Case 2	4	25	20	13	30.39	3.10 s
	5	28	18	24	29.22	5.92 s
	6	30	30	20	27.79	4.52 s

## Data Availability

Data are contained within the article.
